# Profile identification and characterization of risk perceptions and preventive behaviors during the COVID-19 pandemic: A latent profile analysis

**DOI:** 10.3389/fpsyg.2023.1085208

**Published:** 2023-02-20

**Authors:** Yi Xuan Ong, Hye Kyung Kim, Benjamin O. Pelzer, Ying Ying Tan, Wee Ping Lim, Annabelle Kai Lin Chua, Bei Yi Koh

**Affiliations:** ^1^Wee Kim Wee School of Communication and Information, Nanyang Technological University, Singapore, Singapore; ^2^DSO National Laboratories, Singapore, Singapore

**Keywords:** audience segmentation, pandemic communication, risk perception, big five personality traits, health information preferences

## Abstract

In a public health crisis, communication plays a vital role in making sure policies and recommendations from the government level get disseminated accurately to its people and is only considered as effective when the public accepts, supports, complies to, and engages in policies or behaves as per governments’ recommendations. Adopting the multivariate audience segmentation strategy for health communication, this study uses a data-driven analytical method to (1) identify audience segments of public health crisis communication in Singapore based on knowledge, risk perception, emotional responses, and preventive behaviors; and (2) characterize each audience segment according to demographic factors, personality traits, information processing styles, and health information preferences. Results (*N* = 2033) from a web-based questionnaire executed in August 2021 have identified three audience segments: the *less-concerned* (*n* = 650), the *risk-anxious* (*n* = 142), and the *risk-majority* (*n* = 1,241). This study offers insights to how audiences of public health crisis communication perceive, process, and respond to information directed to them during the pandemic, thereby informing policy makers to tailor more targeted public health communication interventions in promoting positive attitude and behavior change.

## 1. Introduction

Since February 2020, the outbreak of COVID-19 has escalated to a global pandemic, with over 500 million cases and 6.28 million deaths to date (World Health Organization, 2022). Due to the nature of transmission and high virality of COVID-19, international health organizations and governments have rolled out preventive measures that include the closure of international and regional borders, lockdowns, and self-isolation. At the individual level, some governments have mandated the adoption of preventive behaviors such as handwashing, mask-wearing, and social distancing to maintain personal hygiene and reduce virus transmission. In such public health crises, communication plays a vital role in making sure policies and recommendations from the governmental level get disseminated accurately to the public. However, dissemination alone does not guarantee that the governments’ recommendations will be followed: Government health advisories are effective only when the public accepts, supports, and complies with the recommendations (Rogers and Storey, 1987; Slater, 1996; Warren and Lofstedt, 2021). Effective health communication should also consider how the public perceives, processes, and responds to information directed in these public health campaigns (Maibach et al., 1996; Slater, 1996). One important tool in understanding the public is audience segmentation (Slater, 1996).

Audience segmentation is the process of grouping audiences into smaller subgroups based on a set of variables that is indicative of an outcome (Slater, 1996). Audience segmentation is a useful communication strategy during a pandemic, as it helps to identify potential problems of current communication efforts for specific audience segments and offers insights to tailor public health communication for these groups (Maibach et al., 1996; Slater, 1996; Maibach et al., 2011). In this vein, the current study aims to capture and identify meaningful clusters of individuals who share similarities in risk attitudes, affective responses, and preventive behaviors during COVID-19. The clusters identified will then be characterized based on demographic and psychological traits, as well as health information preferences and attribution judgments relevant to COVID-19.

While prior research often used a variable-centered approach, which assumes a homogeneous sample, the current study instead adopts a person-centered approach to audience segmentation, using a more data-driven analytical method. This approach further employs Slater’s (1996) multivariate strategy, which enables a selection of variables from multiple theoretical frameworks as determinants of audience segmentation. The results of this study make it possible to uncover the demographic, psychographic, and behavioral characteristics of various audience segments in a population and offer useful insights into tailoring important health information to certain audience segments in order to improve the effectiveness of pandemic communication.

This study takes place in Singapore, a member of G20 with one of the world’s highest vaccination rates (91.4%; Reuters, 2022). Singapore was highly complimented for its crisis response to COVID-19, exemplified with high public trust and compliance to government-recommended preventive measures during the pandemic (Wong and Jensen, 2020; Yeo et al., 2022). Considering the above background, this study has three major objectives. First, this study attempts to identify audience segments on COVID-19 prevention based on Slater’s (1996) multivariate strategy. Specifically, using Latent Profile Analysis (LPA), we employ perceived knowledge, risk perception (inclusive of perceived severity and perceived vulnerability), perceived preventive efficacy, emotional responses, and intention to engage in preventive behaviors as key determinants of audience segments on COVID-19 prevention. Second, this study aims to characterize each identified audience segment regarding its demographic profiles (e.g., age, gender, race), personality traits, and information-seeking and processing styles. Lastly, this study will uncover differences between audience segments on health information preferences and attributions of responsibility in reducing transmission, in the specific context of COVID-19. Results of this study would bring insights to practitioners for tailoring health information to audiences’ characteristics, thus enhancing the effectiveness of communication efforts during a pandemic.

## 2. Literature review

The following review, divided into two sections, offers a critical understanding of the associations between the factors and justifies their use in this audience segmentation study. The first section reviews the overall strategy of audience segmentation and the theoretical explanation of the determinants used for audience segmentation. The second section gives an overview of the two different types of variables associated with audience profile characteristics. The first type focuses on demographic, personality, and behavioral characteristics associated with the audience segmentation determinants. The second type of variables are those that change during a pandemic to understand differences in health information preferences and inform future public health communication strategies.

### 2.1. Audience segmentation strategy—A person-centered approach

Audience segmentation involves the breakdown of a population into smaller clusters of audiences with similar patterns (for instance, in personality, media use habits, or cognitive styles) to achieve positive attitudinal and behavioral change *via* tailored communication efforts (Rogers and Storey, 1987; Grunig, 1989; Slater, 1996). Slater (1996) addresses two major approaches in audience segmentation: (a) the variable-centered approach based on theoretical typologies (Grunig, 1989), and (b) multivariate classification segmentation (one of the person-centered approaches; Maibach et al., 1996). Most extant studies take a variable-centered approach, examining how variables based on a framework (e.g., Risk Perception Attitude (RPA) Framework) relate to demographic factors to uncover segments within the sample (Rimal and Real, 2003; Rimal et al., 2009; Duong, 2022). However, this approach limits meaningful segmentation by treating the sample as homogenous on all other variables not used for segmentation (Slater, 1996; Kleitman et al., 2021). In contrast, the multivariate classification segmentation strategy approach, by acknowledging heterogeneity of all other variables within the sample, enables all variables to be used for a more rigorous and holistic identification of audience segments.

The person-centered approach has been employed in recent studies to examine individual differences on the impacts of COVID-19. For example, Ahmed et al. (2021) segmented the sample population to examine how people adapt to the impacts of COVID-19 based on their stress levels, sleep quality, and adoption of coping activities during a lockdown. Kleitman et al. (2021) also grouped the audience based on their risk attitudes and compliance with preventive behaviors during COVID-19, identifying the characteristics of each audience segment to inform future communication strategies. Similarly, Smith et al. (2021) clustered the population into five classes based on the audience’s intentions to engage in recommended behaviors during the COVID-19 pandemic to identify audiences for targeted messaging. These studies demonstrated the potential utility of a person-centered approach in audience segmentation for offering practical insights into effectively communicating with various segments during a pandemic.

Building on prior works, this study employs a person-centered method to identify subgroups based on shared target behaviors (Smith, 2017). Specifically, this study adopts Slater’s (1996) multivariate classification segmentation strategy, using perceived knowledge, risk perception, perceived preventive efficacy, emotional responses, and behaviors related to a given health domain as determinants of audience segmentation. In doing so, we advance prior literature by leveraging on the audience segmentation to capture differences in health information preferences and attributions of responsibility during the pandemic.

The adoption of preventive behaviors is the key communicative objective during the COVID-19 pandemic. Consequently, this study considered engagement in preventive behavior as a major predictor of audience segmentation. In addition, guided by theory and empirical evidence, we included various psychological factors (i.e., risk perception, perceived knowledge, perceived preventive efficacy, and emotional responses) that are predictive of protective behaviors.

Theoretical frameworks, such as the Health Belief Model (HBM) (Carpenter, 2010) and the RPA Framework (Rimal and Real, 2003), highlight the important roles of risk perception (i.e., perceived vulnerability and perceived severity) and perceived preventive efficacy in predicting preventive behaviors. When individuals have a higher risk perception and think they can engage in preventive behaviors, they are more likely to engage in the behaviors to reduce the risk. Beyond the analytic assessment of risk, affective responses also prominently impact how people interpret the risk at hand (*risk as feeling*, Slovic et al., 2013). Such emotional responses are theorized to influence individuals’ information processing and behavioral actions (Izard, 1993).

Empirically, emotional responses and risk perceptions have been found to predict positive behavioral changes and adherence to government-recommended measures (Weinstein, 1988; Brug et al., 2004; Leung et al., 2004). Kim and Niederdeppe (2013) found that both positive and negative emotional responses were associated with the respondents’ willingness to seek health-related information during the H1N1 pandemic. In the COVID-19 context, Anaki and Sergay (2021) found evidence that perceived severity, perceived susceptibility to COVID-19, and emotional responses predicted preventive behaviors. Kleitman et al. (2021) included risk attitudes and emotional responses toward COVID-19 to identify compliant and non-compliant audience segments to government-recommended measures.

Guided by the HBM and the Social Cognitive Theory (Bandura, 2000), prior works have identified perceived knowledge as a key antecedent of health behavioral intent (Rock et al., 2005; Zhang et al., 2015). As individuals are exposed to certain health information, they tend to retain the information for future evaluation or reference for producing protective behavior when sufficiently motivated (Bandura, 2000; Zhang et al., 2015). For example, Zhang et al. (2015) found that higher perceived knowledge attained from media exposure is associated with higher adoption of preventive behaviors during the H1N1 influenza outbreak. Moreover, the same study demonstrated that media exposure to pandemic information stimulates negative emotions and heightens the perceived knowledge of audiences, resulting in greater adoption of preventive measures (Zhang et al., 2015).

With the theoretical support and empirical evidence of existing literature, this study uses perceived severity, perceived preventive efficacy, and emotional responses experienced in pandemic situations as determinants of audience segments. Moreover, as an extension to Kleitman’s et al. (2021) segmentation study, we take a more holistic approach by including perceived knowledge of the virus (Zhang et al., 2015) and perceived vulnerability to COVID-19 (Duncan et al., 2009) to segment the audiences of COVID-19 communication.

### 2.2. Audience segment characteristics

#### 2.2.1. Demographic traits

Meta-analyzes have highlighted gender and age as key factors directly associated with the adoption of preventive behaviors during pandemics (Bish and Michie, 2010). Specifically, women and older people tend to have greater risk perceptions and thus are more likely to engage in preventive behaviors (Bish and Michie, 2010). However, this meta-analysis did not provide any conclusive results on the association between ethnicity and the adoption of preventive behaviors (Bish and Michie, 2010). Likewise, international surveys have examined various demographic factors to elucidate group differences in risk perceptions related to infectious diseases (de Zwart et al., 2007, 2009). These studies suggested country, age groups, and gender as significant factors that associate with risk perception differences. However, there was still ambiguity from prior works investigating how these demographic factors are linked to preventive behaviors and risk perceptions (de Zwart et al., 2007, 2009; Bish and Michie, 2010). Thus, this study includes race, age, and gender to clarify their associations with audience segments grouped based on preventive behaviors and risk perceptions.

#### 2.2.2. Personality traits

Recent research on COVID-19 has begun to examine how individuals react to pandemic management measures differently depending on their psychological characteristics (Aschwanden et al., 2021). Personality traits can explain how a person thinks, perceives information, feels emotions, and behaves (McCrae and Costa, 2006), including during a pandemic (Aschwanden et al., 2021). In particular, the Big Five personality traits (openness, conscientiousness, extroversion, agreeableness, and neuroticism), introduced by McCrae and John (1992), have been suggested to be associated with health behaviors. For example, individuals who score high in conscientiousness tend to follow rules and regulations, have more self-discipline, and are more likely to adopt more preventive measures (Bogg and Roberts, 2004; Aschwanden et al., 2021; Blagov, 2021). Another trait, neuroticism, reflects the tendency to feel anxious, worried, fearful, or angry in a situation (McCrae and John, 1992). People who score higher in neuroticism tend to experience more negative emotions during the pandemic (Kroencke et al., 2020; Aschwanden et al., 2021). Individuals scoring high in agreeableness show more compliance with rules and regulations (John and Srivastava, 1999; Blagov, 2021; Kleitman et al., 2021). Similarly, individuals scoring high in openness tend to be more resilient, adaptive, and open to the policies set by the government (Aschwanden et al., 2021; Kleitman et al., 2021). Extroverted people tend to be more outgoing, and thus taking preventive measures such as self-isolation and social distancing may be more challenging for them (Aschwanden et al., 2021; Kleitman et al., 2021).

While existing studies include all five personality traits in their analyzes, most of them tend to focus on conscientiousness and neuroticism as these two traits were more significantly associated with adoption of preventive measures (Kroencke et al., 2020; Aschwanden et al., 2021; Blagov, 2021). However, there are inconsistencies in the relationship of each Big Five personality trait with preventive measure adoption or COVID-19-related attitudes. For example, Kleitman et al. (2021) found that conscientiousness and neuroticism did not show any significant differences between compliant and non-compliant groups, differing from previous studies. Likewise, Kleitman et al. (2021) found that non-compliant respondents tended to be more extroverted, in contrast to earlier studies which found that extraversion is associated with taking more precautions (Aschwanden et al., 2021; Blagov, 2021). With inconsistent results from prior works, this study will capture all five traits to clarify and extend findings in relation to personality differences in risk perceptions and preventive behaviors (Sutin and Terracciano, 2016).

#### 2.2.3. Information processing styles—Need for cognition and need for affect

Need for Affect (NFA; Maio and Esses, 2001) and Need for Cognition (NFC; Petty and Cacioppo, 1982; Petty et al., 2009) are suggested to differ across individuals and are associated with the effectiveness of messages in persuasive communication (Haddock et al., 2008; Zhang et al., 2021). NFC and NFA, respectively, can indicate the cognitive and affective orientations which an individual is motivated to engage in when s/he is exposed to a message (Petty and Cacioppo, 1982; Haddock et al., 2008; Zhang et al., 2021). NFA looks at an individual’s tendency to engage in the affective process, relying upon affective information in attitude formation and behavioral intention (Haddock et al., 2008). Individuals with higher levels of NFA tend to be better persuaded when they engage with affect-based (emotionally charged) messaging. In contrast, NFC looks at an individual’s tendency to engage in effortful thinking. Individuals with higher levels of NFC are more likely to be persuaded when exposed to or engaged in cognition-based (informative, factual) messages (Haddock et al., 2008; Petty et al., 2009). Because these information processing styles are closely related to the type of persuasive messaging, it is important to consider how NFA and NFC differ between audience segments and their roles in characterizing the identified segments.

#### 2.2.4. Health information seeking and information preferences during the COVID-19 pandemic

In the face of an ongoing COVID-19 pandemic, communication plays an integral role in managing the outbreak with preventive measures. Individuals often seek information on risks, preventive measures, and symptoms during a pandemic. Health information seeking is affected by various factors including health perceptions, health status, and demographic background (Lambert and Loiselle, 2007; Jacobs et al., 2017; Rayani et al., 2021). Existing studies have shown that engaging in health information seeking can help individuals feel more assured and be more active in adopting preventive measures (Shieh et al., 2010; Rayani et al., 2021). In prior research, time spent on seeking information and the number of pages of health information browsed were associated with higher levels of perceived susceptibility, fear, and anxiety (So et al., 2019). Kleitman et al. (2021) identified that compliant and non-compliant audience segments vary in their preferred information sources, frequency of information seeking, and the tendency to fact-check and compliance. Riding on existing literature, the current study seeks to better understand how the identified audience segments vary in health information-seeking patterns (frequency, time spent, preferred platforms, and intensity) during the pandemic.

Segmentation is the essence of developing a “consumer-oriented” communication (Lefebvre et al., 1995; Maibach et al., 1996, p. 262) that offers insights to policymakers in creating more persuasive messages by understanding their health information-seeking patterns. While current literature examined the health information-seeking patterns and source preferences (Kleitman et al., 2021), there is a research gap on audiences’ content preferences (e.g., topics, content types) during a pandemic, and how such preferences would be associated with audiences’ risk attitudes and behaviors (Avery, 2010; Park et al., 2019). During a pandemic that has persisted for more than 2 years, excessive exposure to health information tends to cause fatigue in the audiences, negatively affecting their attitudes and intention to adopt preventive measures (Ball and Wozniak, 2021). Thus, this study uncovers perceived importance and preferences for different types of COVID-19 information of each audience segment, providing insights for more targeted communication and preventing information fatigue.

#### 2.2.5. Attributions of responsibility

The Attribution Theory (Weiner, 1986) posits that people search for the causes of a negative and unexpected event to make an attribution judgment (Weiner, 1986). An individual or an entity would search for relevant information to evaluate the level of responsibility the stakeholder has in an event or a crisis (Fiske and Taylor, 1991). The attribution of responsibility can be also influenced by various factors, such as the preference of information sources (Park and Lee, 2016), risk perceptions and crisis emotions (Kim and Niederdeppe, 2013), and political orientation (Zhang et al., 2021). The inclusion of this variable allows the examination of how segments of the audience attribute responsibility to different stakeholders (citizens, government, healthcare workers, schools, and workplaces) in reducing transmission during the COVID-19 pandemic. Understanding how an individual attributes responsibility during COVID-19 can inform policymakers on how to develop favorable responses and manage public opinion on the crisis (Coombs, 2016).

## 3. Methods

### 3.1. Sampling and data collection

This study was conceptualized and executed in Singapore, during the second global wave of the pandemic, where the Delta variant was the main variant of infection in Singapore. Participants were recruited with panels managed by local market research firms. We employed quota sampling according to Singapore’s population census in 2020 (Singapore Department of Statistics, 2021) to ensure demographic representativeness of the sample in terms of gender, age, and ethnicity. Only Singaporeans citizens aged 18 and above were eligible to take part in the study. The final sample consisted of 2,033 participants, including 1,038 (51.1%) females and 995 (48.9%) males. The sample consists of 266 (13.1%) respondents in the age group of 18–24, 361 (17.8%) in the 25–34 age group, 399 (19.6%) aged 35–44, 409 (20.1%) respondents aged 45–54, 379 (18.6%) from the 55–64 age group, and 219 (10.7%) respondents who are aged 65 and above. The sample’s racial distribution is representative of Singapore’s racial distribution, with 1,509 (74.2%) Chinese, 268 (13.2%) Malays, 197 (9.7%) Indians, and 59 (2.9%) respondents who identified as Eurasian or other ethnic groups. Participants recruited from the local market research agency were compensated at the discretion of the market research agency.

### 3.2. Measures

#### 3.2.1. Determinants of audience segmentation

We assessed the following nine measures as determinants of the audience segmentation: preventive behaviors (nine items, Caress et al., 2010; 4-point scale, 1 = never to 4 = always, e.g., mask-wearing, handwashing, social distancing, *M* = 30.57, SD = 3.98); perceived knowledge (four items, Zhang et al., 2015; 7-point Likert scale, 1 = strong disagree, 7 = strongly agree, e.g., “I am knowledgeable about COVID-19.”; *M* = 5.21, SD = 1.01); perceived severity (three items, Rimal and Real, 2003; 7-point Likert scale, 1 = strong disagree, 7 = strongly agree, e.g., “COVID-19 is a deadly disease,” *M* = 5.25, SD = 1.31); perceived preventive efficacy (two items, Rimal and Real, 2003; 7-point Likert scale, 1 = strong disagree, 7 = strongly agree, e.g., “I can prevent myself from contracting COVID-19,” *M* = 5.12, SD = 1.31); perceived vulnerability (one item, Duncan et al., 2009; 5-point scale, 1 = extremely unlikely, 5 = extremely likely, “If you do not take any preventive actions against COVID-19, how likely do you think you may contract the virus within 3 months?”); emotional responses (nine items, Izard, 1977; Fredrickson et al., 2003; 5-point scale, 1 = never, 5 = all the time, e.g., “grateful” (four discrete positive emotions, *M* = 3.67, SD = 0.80), “anxious” (four discrete negative emotions, *M* = 3.05, SD = 0.95), and “sympathy,” *M* = 3.80, SD = 0.96). Responses from each variable were averaged to form their respective score.

#### 3.2.2. Characteristics of audience segments

##### 3.2.2.1. Demographic and personality traits

In addition to demographic traits such as age, gender, and race, we measured the Big Five personality traits and need for cognition and affect. Using the full 50-item scale of the Big Five Personality Traits (Goldberg, 1992), participants were asked to rate the extent to which they agree with each statement measuring their openness to experience, conscientiousness, extraversion, agreeableness, and neuroticism, on a scale from 1 (disagree) to 5 (agree). Responses from each variable were averaged to form its respective score.

##### 3.2.2.2. Information processing styles

Using the ten-item measurement derived from Cacioppo et al. (1984), respondents indicated the extent of their need for cognition on a scale of −3 (strongly disagree) to 3 (strongly agree). We measured the need for affect with a ten-item scale derived from Maio and Esses (2001), for which respondents needed to indicate how similar the person in each statement is to the respondent from 1 (not like me at all) to 6 (very much like me). Responses from each variable were averaged to form its respective score.

##### 3.2.2.3. Health information seeking

From a selection of 15 platforms provided (Majid and Rahmat, 2013), we asked the respondents to rank their top three preferred platforms they use when they want to find out health information. Based on their top three preferences, we asked the respondents to indicate the frequency of use for health information search in the past week on a 4-point scale (1 = never, 4 = often) and the duration spent in a day on average for searching health-related information on a 6-point scale (1 = less than 10 min, 6 = more than 3 h) (Ayers and Kronenfeld, 2007). Similarly, the intensity of the top three platform use was measured on a 5-point Likert scale (1 = strongly disagree, 5 = strongly agree) with four items derived from Ellison et al. (2007) (e.g., “This platform has become part of my daily routine”). Responses from each variable were averaged to form the respective scores.

##### 3.2.2.4. Health information preferences

There are three variables measured for health information preferences. First, we asked the respondents to rank five out of 17 content types found in health news coverage in order of importance from 1 (most important) to 5 (least important). The items were derived from Majid and Rahmat (2013) and Wong and Sam (2010). Second, on a scale from 1 (most likely) to 6 (least likely), we asked the respondents to rate their likelihood of reading about news reporting from a selection of six news frame categories derived from Shih et al. (2008). Lastly, we derived nine news content types from Quandt et al. (2020) and asked the respondents to rank the top five topics in order of preference, with 1 being the most liked topic.

##### 3.2.2.5. Attribution of responsibility

We derived the scale for attribution of responsibility from Kim and Niederdeppe (2013). The respondents were asked to indicate on a scale from 1 (not at all responsible) to 7 (totally responsible), the level of responsibility each of the five actors should bear for reducing the transmission of COVID-19 among the population (e.g., government, citizens, schools, healthcare workers, and workplaces).

### 3.3. Statistical analysis

A total of three analytic steps are followed to achieve the three research objectives. First, the Latent Profile Analysis (LPA), a method of the person-centered approach, is used to identify potential audience segments of individuals who share similar patterns of risk perception, perceived knowledge, perceived preventive efficacy, emotional responses, and preventive behaviors during COVID-19. To enable direct comparisons, variables were standardized before executing LPA with *mclust* on statistical software package Rstudio (Scrucca et al., 2016). The LPA classified the observations in a dataset into a set of profiles based on their homogenous characteristics across the set of estimated values of the determinants. The results of LPA offered a systematic grouping of the units of analysis based on the clustering of observations using standardized means. This data-driven approach allows researchers to assume heterogeneity within the sample while achieving conceptual validity with the use of widely explored variables in risk and health communication research as predictors of the audience profiles. Second, to characterize and examine differences between the identified audience segments in demographic factors, personality traits, information consumption, and information processing style, crosstab analysis with Chi-square tests were performed on categorical variables. We also performed a series of ANOVA tests, with Bonferroni post-hoc tests, on ordinal and continuous variables. Finally, to investigate the attribution of responsibility and health information preferences of different audience segments, a series of ANOVA tests were performed. The statistical software package IBM SPSS (Statistical Package for Social Sciences) V23.0 was used for the later analyzes testing for profile differences.

## 4. Results

### 4.1. Latent profile analysis

#### 4.1.1. Model selection

LPA was performed for 2–6 class solutions, with 1 class as the default. Table 1 illustrates the model fit statistics and the proportion of class membership of 2–6 class solutions. To select the number of classes, this study inspected the Bayesian Information Criterion (BIC), the Integrated Completed Likelihood Criterion (ICL), and the Bootstrap Likelihood Ratio Test (BRLT), which is assessed based on the value of p associated with the log of this likelihood ratio (Nylund et al., 2007; Achterhof et al., 2019). Since the BRLT *p*-values are all less than 0.001 in all class solutions, BIC and ICL values were inspected for class selection. The BIC and log-likelihood values illustrated the trend that solutions with higher number of classes proved better model fit, with the 5-class solution having the greatest BIC value (Achterhof et al., 2019; Wardenaar, 2021). However, based on the ICL value, which penalizes solutions with classification uncertainty, the 3-class solution (bolded in Table 1) performed the best (Biernacki et al., 2000; Bertoletti et al., 2015).

**Table 1:** Latent profile analysis model fit statistics and class membership.

Classes in the model	BIC	ICL	LogL	df	BRLT_*p*	Class 1	Class 2	Class 3	Class 4	Class 5	Class 6
2-Class	−43423.99	−44257.18	−21506.33	52	<0.001	734	1,299				
3-Class	**−43218.47**	**−44100.29**	**−21365.48**	**64**	**<0.001**	**650**	**142**	**1,241**			
4-Class	−43175.88	−44269.53	−21306.1	74	<0.001	148	713	1,007	165		
5-Class	−43109.70	−44174.56	−21234.92	84	<0.001	135	563	1,034	158	143	
6-Class	−43146.30	−44568.25	−21215.14	94	<0.001	248	145	978	258	285	119

**Figure 1:**
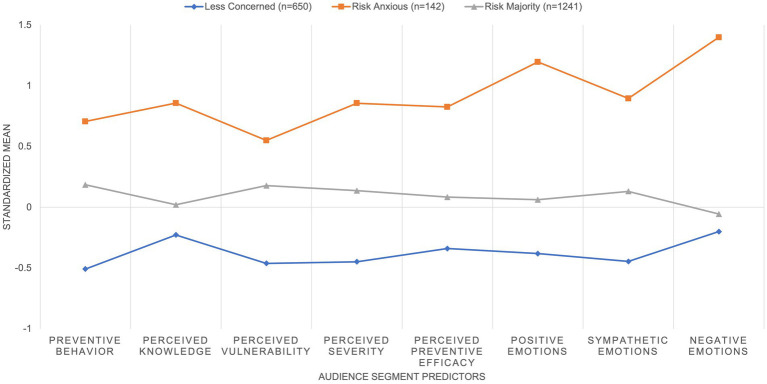
3-class solution from LPA on audience segmentation.

Figure 1 presents the audience segmentation based on standardized mean scores for each of the three classes on the eight variables included in LPA. We confirmed that the three classes were significantly different from each other in all eight determinant variables using one-way ANOVA with Bonferroni post-hoc tests. Inspired by the RPA framework which posits four different groups of audiences based on their risk perception and efficacy beliefs to act on certain health-related actions (Rimal and Real, 2003), this study names the respective class based on its scores on the key determinants. Based on the 3-class profile selected, Class 1 (*n* = 650), consisting of 32% of the sample, is profiled to least engage in preventive behavior and scored the lowest for risk perceptions and emotional responses. They have the lowest mean scores of all the predictor measures related to risk perception and preventive behavior during COVID-19. Therefore, this group appeared to be *less-concerned* about COVID-19, having a lower likelihood to engage in preventive measures such as washing hands, wearing masks, and social distancing. They also appeared to feel positive and negative emotions less often as compared to other classes.

Class 2 consists of the majority, with 61% of the sample (*n* = 1,241). Class 2 members are respondents who scored around the mean of determining variables, scoring only slightly higher than Class 1 members on perceived subjective knowledge on COVID-19, and the tendency to feel negative emotions during the COVID-19 pandemic. Class 2 members appeared to engage in preventive measures more actively than Class 1 members and perceived that they would be more likely to contract COVID-19 if they do not take any preventative measures, as compared to Class 1 members. However, they scored lower than Class 3 in the mean scores of all predictor variables. With Class 2 having mean scores that hover near the mean scores of all the measures used for predicting profiles in the LPA, this class is labeled as the *risk-majority*.

Class 3 (*n* = 142), consisting of 7% of the sample, is the group that equips itself with knowledge, scoring the highest out of the three classes in the mean scores of risk perception and preventive behaviors. They tend to feel crisis emotions (both positive and negative) more often during the pandemic. Thus, they are labeled as *risk-anxious* toward COVID-19.

### 4.2. Results for audience segment characteristics

Differences are observed between the identified audience segments in Age, Gender, and Race (Table 2). The *risk-majority* and the *risk-anxious* have a higher proportion of the female audience, following the gender distribution of the overall sample. The *less-concerned* profile differs from the other two audience segments with a higher proportion of male audience (53.7%) (male _risk-majority_ = 46.3%, male _risk-anxious_ = 48.6%), and the overall sample (48.8%). Looking at the audience in each age group, all audience segments generally follow the age distribution of the overall sample. The *risk-anxious* profile has a significantly lower proportion of the younger population (respondents in age groups 18–24 and 24–34) and a higher proportion of respondents aged between 55 and 74, who tend to face greater vulnerability against COVID-19 and perceive COVID-19 to be more severe, as compared to other classes and the overall sample. While the *risk-majority* members’ age profile follows that of the overall sample, the *less-concerned* members appear to have a higher proportion of respondents aged 25–34 as compared to members of the other segments and the overall sample. On the racial distribution of profiles, all audience segments show similar racial distribution as the overall sample, which is representative of Singapore’s racial distribution. However, the *risk-anxious* racial distribution differs slightly from the general racial distribution, with only 54.2% Chinese, 26.1% Malays, and 19.7% of Indian and other ethnic groups (e.g., Eurasian, Filipinos).

**Table 2:** Demographic differences between profiles.

Variable		Overall		Class 1		Class 2		Class 3	
		*n*	%	*n*	%	*n*	%	*n*	%
Demography									
Gender	Male	993	48.8%	349	53.7%	575	46.3%	69	48.6%
	Female	1,038	51.1%	300	46.2%	665	53.6%	73	51.4%
Age	18–24	266	13.1%	87	13.4%	169	13.6%	10	7.0%
	25–34	361	17.8%	128	19.7%	220	17.7%	13	9.2%
	35–44	399	19.6%	138	21.2%	230	18.5%	31	21.8%
	45–54	409	20.1%	133	20.5%	253	20.4%	23	16.2%
	55–64	379	18.6%	110	16.9%	236	19.0%	33	23.2%
	65–74	206	10.1%	52	8.0%	122	9.8%	32	22.5%
	75+	13	0.6%	2	0.3%	11	0.9%	0	0.0%
Race	Chinese	1,509	74.2%	481	74.0%	951	76.6%	77	54.2%
	Malay	268	13.2%	76	11.7%	155	12.5%	37	26.1%
	Indian	197	9.7%	72	11.1%	105	8.5%	20	14.1%
	Eurasian	10	0.5%	6	0.9%	3	0.2%	1	0.7%
	Filipino	18	0.9%	2	0.3%	11	0.9%	5	3.5%
	Others	23	1.1%	11	1.7%	12	1.0%	2	1.4%
	Undisclosed	8	0.4%	4	0.6%	4	0.3%	0	0.0%

Across the five personality traits, Extraversion and Neuroticism exhibited the lowest average score, while Conscientiousness exhibited the highest average (Table 3). Out of the Big Five Personality Traits, only Neuroticism does not show statistically significant differences between the audience segments. When compared pairwise, the *less-concerned* and the *risk-majority* profiles do not show significant differences from each other for Openness and Conscientiousness, while all classes differ from each other significantly for Extraversion and Agreeableness. Compared to the other two classes, the *risk-anxious* respondents score significantly higher on Openness, Conscientiousness, Extraversion and Agreeableness. The *less-concerned* respondents scored the lowest on Extraversion and Agreeableness as compared to the other two segments.

**Table 3:** Differences in big five personality traits and information processing styles.

Variable	Overall		Class 1		Class 2		Class 3		*F*(2, 2030)	^a^Mean difference (Class 1–2)	^a^Mean difference (Class 1–3)	^a^Mean difference (Class 2–3)
	*M*	SD	*M*	SD	*M*	SD	*M*	SD				
Openness	33.36	5.46	33.04	6.28	33.32	4.97	35.18	5.24	9.11***	0.27	2.14***	1.87***
Conscientiousness	37.04	5.45	36.72	6.15	37.02	5.02	38.70	5.36	7.77***	0.30	1.98***	1.68**
Extraversion	28.55	6.68	27.52	7.52	28.78	6.24	31.27	5.24	20.59***	1.26***	3.75***	2.49***
Agreeableness	36.44	5.60	35.15	6.50	36.87	4.95	38.66	5.20	33.18***	1.72***	3.51***	1.79**
Neuroticism	28.07	7.62	28.16	8.47	27.88	7.09	29.33	7.93	2.39^ns^	0.28	1.17	1.45
Need for cognition	33.16	5.66	32.82	6.27	33.22	5.35	34.25	5.22	3.85*	0.40	1.43*	1.03
Need for affect	4.78	7.38	4.10	8.13	5.11	7.00	5.08	6.86	4.16*	1.01*	0.99	0.03

^a^Pairwise comparison/post-hoc executed with Bonferroni. *Shows *p* < 0.05, ***shows *p* < 0.001; ^ns^means non-significant.

There was a significant difference between the *less concerned* and the *risk-anxious* profiles on the need for cognition, where the latter scored significantly higher for the need for cognition than the former segment (Table 3). No class difference is observed between the other two comparisons. In contrast, there is a significant difference between the *risk-majority* and the *less-concerned* profiles, where the former scored significantly higher than the latter on the need for affect. No statistically significant difference is found between the other two pairwise comparisons.

Different health information-seeking behaviors during the pandemic are observed among the three identified audience segments (Table 4). The *risk-anxious* is found to have the highest frequency of health information seeking in a week (*M* = 3.52 out of 4). On the other hand, the *less-concerned* has the lowest frequency of health information seeking (*M* = 2.97 out of 4), while the *risk-majority* has the mean frequency that hovers close to that of the overall sample. In terms of the duration of health information seeking, the *risk-anxious* is found to spend about 10–60 min each day, the longest among the three classes of respondents (*M* = 2.51). However, no statistically significant difference is observed between the other two segments in the average time spent per day. Group differences are also observed between all three audience segments in health information platform intensity, with the *risk-anxious* having the highest intensity, followed by the *risk-majority*, and the *less-concerned* having the least intensity on health information.

**Table 4:** Class comparison on health information seeking.

Variable	Overall		Class 1		Class 3		Class 2		*F*(2, 2030)	^a^Mean difference (Class 1–3)	^a^Mean difference (Class 1–2)	^a^Mean difference (Class 2–3)
	*M*	SD	*M*	SD	*M*	SD	*M*	SD				
Seeking frequency	3.13	0.70	2.97	0.79	3.16	0.46	3.52	0.50	41.87***	0.19***	0.55***	0.36***
Seeking duration	1.94	0.99	1.83	0.98	1.93	0.96	2.51	1.18	28.50***	0.11	0.68***	0.58***
Platform intensity	3.30	0.86	3.07	0.92	3.33	0.79	4.05	0.72	82.59***	0.26***	0.97***	0.71***

^a^Pairwise comparison/post-hoc executed with Bonferroni. ***Indicates *p* < 0.001.

Out of the 15 platform choices for seeking health information on COVID-19, differences in platform preferences between the three audience segments can be seen in the following eight platforms (Table 5). The three audience segments shared similarities in their preference for formal sources of information. Official government websites, newspapers, and TV are the top three preferred platforms for seeking COVID-19 information for most of the *less concerned* and the *risk-majority* audiences. The *risk-anxious* preferred the TV, official government websites, and official government social media sites. However, the *less concerned* segment is found to have the highest proportion of respondents preferring informal sources (e.g., personal messaging applications, online forums, independent blogs) as compared to the other two segments and the overall sample.

**Table 5:** Differences in platform preferences.

Variable	Overall		Class 1		Class 2		Class 3	
	*n*	%	*n*	%	*n*	%	*n*	%
Official government websites	1,183	58.2%	352	54.2%	757	61.0%	74	52.1%
Official government social media	617	30.3%	250	38.5%	568	45.8%	68	47.9%
Newspaper	748	36.8%	263	40.5%	448	36.1%	37	26.1%
TV	852	41.9%	251	38.6%	519	41.8%	82	57.7%
Radio	279	13.7%	75	11.5%	170	13.7%	34	23.9%
Personal messaging applications (e.g., WhatsApp, Telegram, Line)	126	6.2%	53	8.2%	71	5.7%	2	1.4%
Online forums (e.g., HardwareZone, Reddit)	94	4.6%	45	6.9%	45	3.6%	4	2.8%
Independent blogs (e.g., Mothership)	172	8.5%	83	12.8%	84	6.8%	5	3.5%

Due to the nature of a ranked data measure for information preferences, this study executed nonparametric independent-samples Kruskal-Wallis’s test, with Bonferroni correction for pairwise comparisons (Table 6). Out of 17 listed content types, only seven indicated differences between the three audience segments for content type importance. The *less-concerned* and the *risk-majority* audiences showed differences in their topic importance for information on “the updated list of countries affected by COVID-19.” The *less-concerned* audience segment only differed from the *risk-anxious* on topic importance for information on “the current and future pandemic plans in Singapore.” The *risk-majority* and the *risk-anxious* differed in their topic importance on “the updated information of cluster areas in Singapore.” The *risk-anxious* audiences indicated differences from the other two segments in their perceived importance for information on “the spread of the outbreak in Singapore,” “information on proper procedures of washing hands,” “information on proper ways for putting on a mask,” and “the updated number of recovered patients infected by COVID-19 in Singapore.”

**Table 6:** Preference for COVID-19 information.

Variable	Overall		*χ*^2^ (2, 2030)	*p*	^a^Mean difference (Class 1–2)	^a^Mean difference (Class 1–3)	^a^Mean difference (Class 2–3)
	*M*	SD					
Prevention and control of COVID-19	0.32	0.39	3.88	0.144			
COVID-19 signs and symptoms	0.19	0.32	0.35	0.840			
Spread of COVID-19 in Singapore	**0.46**	**0.41**	**14.06**	**0.001**	**45.134** ^**ns**^	137.330*	182.464***
Availability of treatments for COVID-19 and its side effects	0.17	0.29	3.82	0.148			
Government’s advice for individuals having COVID-19-like symptoms	0.18	0.30	5.01	0.082			
Information about proper procedure for washing hands	**0.04**	**0.17**	**10.76**	**0.005**	**2.777** ^**ns**^	74.959**	72.182**
Information about proper ways for putting on a mask	**0.05**	**0.18**	**9.93**	**0.007**	**.709** ^**ns**^	78.816**	79.524**
COVID-19 vulnerable groups and the level of risk	0.12	0.26	2.40	0.301			
COVID-19 protection products and their availability at major retail outlets	0.06	0.19	1.18	0.556			
Updated information about COVID-19 cluster areas in Singapore	**0.45**	**0.39**	**7.03**	**0.030**	**25.454** ^**ns**^	**106.817** ^**ns**^	132.271*
Updated information about current and future pandemic plan for Singapore	**0.31**	**0.36**	**6.16**	**0.046**	**32.084** ^**ns**^	125.347*	**93.263** ^**ns**^
Procedure for seeking treatment of suspected COVID-19 patients at clinics or hospitals	**0.10**	**0.23**	**0.10**	**0.951**			
Updated list of COVID-19-affected countries	**0.10**	**0.23**	**8.85**	**0.012**	55.748*	**1.652** ^**ns**^	**57.4** ^**ns**^
Updated number of COVID-19 fatalities in Singapore and other countries	0.16	0.29	4.81	0.090			
Updated number of COVID-19-infected cases across the world	0.13	0.27	1.82	0.403			
Origin of COVID-19 virus	0.04	0.15	4.59	0.101			
Updated number of COVID-19-infected patients who have recovered in Singapore	**0.11**	**0.26**	**14.84**	**0.001**	**5.511** ^**ns**^	143.068***	137.557***
Consequences	**0.59**	**0.24**	**11.75**	**0.003**	**62.099** ^**ns**^	173.720**	**111.622** ^**ns**^
Uncertainty	0.46	0.24	3.73	0.155			
Action	0.63	0.23	1.17	0.557			
Reassurances	**0.42**	**0.25**	**11.39**	**0.003**	**30.409** ^**ns**^	181.879**	151.470**
Conflict	**0.35**	**0.24**	**10.10**	**0.006**	83.140**	**111.349** ^**ns**^	**28.209** ^**ns**^
New evidence	**0.55**	**0.24**	**10.68**	**0.005**	**38.687** ^**ns**^	175.819**	137.132*
Statistical information about the current progress and spread of COVID-19	**0.52**	**0.40**	**9.89**	**0.007**	85.617**	**27.028** ^**ns**^	**58.589** ^**ns**^
Factual recounts and reports of the historical development and current trends of COVID-19	0.25	0.34	0.15	0.929			
Anecdotal perspectives of past and current trends of COVID-19	0.11	0.24	5.89	0.052			
Scientific insights and expert opinions related to COVID-19	0.35	0.37	2.20	0.333			
International affairs and developments related to COVID-19	0.32	0.34	2.04	0.360			
Policy developments and social support in Singapore	**0.37**	**0.36**	**7.89**	**0.019**	**45.661** ^**ns**^	**82.307** ^**ns**^	127.697*
Economic effects and trends of COVID-19	0.43	0.36	0.84	0.657			
Social and public life consequences of COVID-19	0.43	0.36	1.69	0.430			
Content related to human interest	0.21	0.31	1.68	0.431			

^a^Pairwise comparison/post-hoc executed with Bonferroni. *Indicates *p* < 0.05, ***indicates *p* < 0.001, ^ns^means non-significant. Bolded values indicate statistically significant items.

For reported news on COVID-19 that audiences are likely to read, all six news frame categories except for content related to “uncertainty of the disease” and “actions taken against COVID-19,” showed significant differences between the three audience segments. *Risk-anxious* audiences differ from the other audiences in their likelihood to read news reporting on “consequences of the disease,” “reassurances” and “new evidence on the disease.” The *less-concerned* and the *risk-majority* differ in their likelihood to read news reporting “conflicts on opinions on the pandemic,” but no other differences were identified from other pairwise comparisons.

Out of the nine content types that were provided, the audiences’ most liked topic was “statistical information about the current progress and spread of COVID-19″. This was followed by “the economic effects and trends of COVID-19”, “social and public life consequences of COVID-19”, “policy developments and social support in Singapore,” “scientific insights and expert opinions related to COVID-19,” and “international affairs and developments related to COVID-19.” However, only topics related to statistical information and policy developments showed statistically significant differences between the risk profiles. The difference is only observed for news based on statistical information between the *less-concerned* and the *risk-majority*. Difference between profiles is also observed between *risk-majority* and *risk-anxious* segments on the news related to “policy developments and social support in Singapore.” Other profile differences were not observed.

Out of the five stakeholders involved, health workers were attributed with the lowest responsibility, followed by schools and the workplace (Table 7). Citizens and government were attributed with the highest level of responsibility in reducing transmission during the pandemic. The *less-concerned* segment indicated a greater variance in their responsibility attribution, with the highest attributed to the government and the lowest to the health workers. On the other hand, the *risk-anxious* attributed the greatest extent of responsibility to all stakeholders, all scoring above 6.5 out of 7, with the greatest being the government, and the lowest being schools and the workplace. The *risk-majority* was more varied in the attributions of responsibility, showing a similar pattern to that of the *less-concerned* segment. Instead of attributing the government with the highest level of responsibility, *risk-majority* audiences attributed it to the citizens for reducing the transmission of COVID-19. Results based on ANOVA showed a significant difference between the three segments in the responsibility attributed to citizens, healthcare workers, and schools. However, significant differences were observed between the *risk-anxious* and the other two segments for responsibility attributed to the government and workplace, but not between the *less-concerned* and the *risk-majority* for these two entities. Thus, it can be inferred that the *risk-anxious* attributes more responsibility to the government and workplace in reducing the transmission of COVID-19.

**Table 7:** Attributions of responsibility by each audience segment.

Variable	Overall		Class 1		Class 2		Class 3		*χ*^2^ (2, 2030)	^a^Mean difference (Class 1–2)	^a^Mean difference (Class 1–3)	^a^Mean difference (Class 2–3)
	*M*	SD	*M*	SD	*M*	SD	*M*	SD				
Citizen	6.25	1.05	6.07	1.28	6.31	0.92	6.60	0.83	19.74	0.240***	0.531***	0.291**
Government	6.25	0.99	6.19	1.13	6.23	0.92	6.65	0.79	12.97	0.04^ns^	0.459***	0.416***
Health worker	5.92	1.21	5.73	1.37	5.94	1.13	6.60	0.84	31.01	0.209**	0.865***	0.656***
School	5.94	1.15	5.79	1.32	5.95	1.06	6.58	0.81	28.64	0.158**	0.795***	0.637***
Workplace	6.02	1.08	5.93	1.21	6.00	1.02	6.58	0.79	22.34	0.07 ^ns^	0.657 ^ns^	0.583 ^ns^

^a^Pairwise comparison/post-hoc executed with Bonferroni. *Indicates *p* < 0.05, **indicates *p* < 0.01, ***indicates *p* < 0.001, ^ns^means non-significant.

## 5. Discussion

The current study is one of the first that adopted Slater’s (1996) recommended strategy for multivariate audience segmentation. This study extends recent works on audience segmentation, such as Kleitman et al. (2021) and Smith et al. (2021) which clustered the sample primarily based on the engagement of preventive measures in COVID-19. Specifically, we took a more holistic approach by segmenting audiences based on their perceived knowledge, risk perceptions, and emotional responses, besides preventive behaviors during the COVID-19 pandemic.

Taking the person-centered approach, this study identifies and segments the sample into three main audience profiles: the *less-concerned* (32%), the *risk-majority* (61%), and the *risk-anxious* (7%). This study mirrors prior audience segmentation studies whereby people showcase complexity in patterns of behavior adoption. However, the current study brings originality in comparison with prior works. Instead of the identified traits of the resultant compliant and non-compliant audience segments in Kleitman’s et al. (2021), results of the current study identified three clusters of varying risk attitudes during the pandemic in a highly complaint sample. The results also differ from Smith et al. (2021), who identified five different audience classes based on their likelihood in engaging their preferred preventive measures, namely––the adherents, social distances, hygiene stewards, symptom managers and refusers, suggesting targeted campaigns for specific segments. In addition to characterizing traits of each identified audience segment, this study also highlighted the media and information preferences of each audience segment. Our finding shows that the *less-concerned* and the *risk-anxious* segments differ the most in perceived severity of COVID-19 and emotional responses felt during COVID-19. Possibly due to lower perceived severity and less emotional responses, the *less-concerned* also reported lower adoption of preventive behaviors compared to the *risk-anxious* segment.

### 5.1. Differences in audience segment characteristics

The three profiles differ from each other on several factors, including demographic characteristics, personality traits, information processing styles, health information seeking and preferences, and attributions of responsibility.

#### 5.1.1. Demographic differences

Reflecting their highest risk perception and adoption of preventive measures, the *risk-anxious* consists of the highest proportion of respondents aged between 55 and 74. This finding is in line with recent studies which suggested that perceptions of COVID-19 severity increase with age (de Zwart et al., 2009; Bruine de Bruin, 2021; Rosi et al., 2021). Individuals in this age group perceive themselves to be more vulnerable to the complications brought by a COVID-19 infection due to pre-existing health conditions. Therefore, they tend to perceive greater severity and vulnerability toward COVID-19 and are more likely to engage in preventive measures (Bish and Michie, 2010).

On the other hand, gender differences in risk perception and adoption of preventive measures between segments corroborate with existing studies (Brug et al., 2004; Leung et al., 2004; Bish and Michie, 2010). Females are associated with higher perceived severity and higher adoption in preventive measures, as seen in the higher female audience proportion in *risk-majority* and *risk-anxious* segments. The *less-concerned* profile also consists of a higher proportion of male respondents. In terms of ethnicity, the *risk-anxious* indicated a higher proportion of Malays, Indians, and other ethnic groups in Singapore, as compared to other segments. This result could be influenced by other ethnic characteristics, and further studies should consider clarifying the association of ethnicity with risk perception and preventive measure adoption.

#### 5.1.2. Personality differences

Out of the five personality traits, only neuroticism did not have any significant group differences, possibly due to the specific pandemic context that involves worry and anxiety. The *risk-anxious* audiences are found to be more open, conscientious, extraverted, and agreeable as compared to the *less-concerned* and the *risk-majority*. This finding suggests that the more extroverted respondents are more compliant with preventive measures, which counters the findings of Kleitman et al. (2021). Other personality traits like agreeableness, conscientiousness, and openness, in combination with extraversion, may exert an influence that delays gratification of social activities and adopt preventive measures (Aschwanden et al., 2021). The high scores in agreeableness, conscientiousness, and openness show that the *risk-anxious* audiences are more adaptable to changes in safety management measures and adhere to the regulations designed to reduce the transmission of COVID-19. Conversely, the *less-concerned* are shown to be less extraverted and agreeable than the *risk-anxious* and the *risk-majority*. Identifying these personality traits would be helpful for future campaigns when reaching out to people profiled under similar characteristics.

#### 5.1.3. Information processing styles

Group differences are observed only between the *less-concerned* and the *risk-anxious* for NFC, consistent with Xu and Cheng’s (2021) findings on the association of NFC with engagement of preventive measures. That is, higher NFC is associated with enhanced risk perception, and higher adoption of preventive measures (Lachlan et al., 2021; Xu and Cheng, 2021). The higher scores of NFC in the *risk-anxious* audience segment also highlighted that this segment tends to use effortful, systematic information processing as compared to heuristic reasoning (Petty et al., 2009). The *risk-majority* scored significantly higher for NFA than the *less-concerned* and the *risk-anxious*. This shows that most of the audience are more likely to be persuaded by more emotionally charged messaging (Haddock et al., 2008). The *less-concerned* audience segment has low NFC and NFA as compared to other audience segments, which may prove to be a challenge for practitioners in making positive changes for this particular audience segment.

#### 5.1.4. Differences in health information seeking and preferences

The *risk-anxious* differs from the other two profiles, with the highest frequency, and the longest time spent per day for health information seeking during COVID-19. A greater amount of time could be spent on fact-checking (Kleitman et al., 2021), given that they are identified as those who are more likely to perceive vulnerability to the disease (Brug et al., 2004; Bish and Michie, 2010; Bruine de Bruin, 2021). The *less-concerned* audiences are found to have lower frequency and intensity in seeking health information. Future studies should examine the motivations of each audience segment on their use of certain platforms for health information seeking during the pandemic.

Noteworthy findings related to health information preferences are observed. All three profiles reported a strong preference for official governmental sources and mainstream media for health information during the pandemic. This result similar to previous studies where the compliant group of respondents reported greater use of official sources, suggesting that respondents rely on credible sources for health information during the pandemic (Kleitman et al., 2021; Lwin et al., 2021). However, the *less-concerned* respondents also reported a significantly higher preference for informal sources like online forums, blogs and personal messaging applications as compared to the other two segments. This finding is in line with the concerns that previous studies have pointed out (Kleitman et al., 2021; Lwin et al., 2021). The use of both formal and informal sources for health information seeking during a pandemic would affect, positively and negatively, the effectiveness of campaigns used for managing the pandemic (Kleitman et al., 2021).

Each audience segment is found to have varied health information preferences. The *risk-anxious* segment indicated topics related to the spread of the pandemic and the proper procedures of preventive measures as important. This could be due to the higher perceived severity and vulnerability to COVID-19 and adoption levels of preventive behaviors found in the *risk-anxious*, thereby perceiving more importance should be placed on these topics to better manage the pandemic. Similar emphasis should also be placed on different topic preferences when tailoring messages for different clusters insofar as significant differences are observed between the *risk-anxious* and the *risk-majority*. Due to the progression of the pandemic situation in Singapore, updated information on clusters may not be seen as important by the *risk-majority* as compared to the *risk-anxious*. Further, the *risk-anxious* audiences are more likely to read stories reporting consequences of COVID-19, new evidence related to COVID-19, and reassurances on COVID-19. This could be associated to the risk perceptions and other characteristics of this audience segment. Moreover, the likelihood to read stories reporting reassurances can be associated with the higher frequency of positive emotions felt by the *risk-anxious* during the pandemic.

The *less-concerned* audience differed significantly from the *risk-majority*. They indicated greater importance on the “updated list of countries affected by COVID-19” and less importance on “current and future pandemic plans.” This difference could be indicated by personality traits characterizing the *less-concerned*, where they are less extroverted and less open, which would mean they are less open to changing policies or updates on the situation. In addition, the *less-concerned* segment indicated a greater likelihood to read conflict stories focusing on clash in opinions about COVID-19. This could be due to their preference of seeking health information on informal platforms such as blogs and online forums (Lachlan et al., 2021), or the need for stimulation from the infodemic/fatigue of prolonged exposure to COVID-19 information.

#### 5.1.5. Differences in attributions of responsibility

The results have identified differences in responsibility attribution from each audience segment. The *risk-anxious* differs from the other two audience segments by attributing higher crisis responsibility to all stakeholders (citizens, government, health workers, school, and workplace) in reducing the transmission of COVID-19 in Singapore. This result could be linked to emotional reactions in this segment considering positive associations reported in prior work between attributions of responsibility and frequency of positive and negative emotions felt during a pandemic (Kim and Niederdeppe, 2013; Wong et al., 2021). The high responsibility attributed to all stakeholders by the *risk-anxious* audiences may not only be associated with the severity of the pandemic (Coombs, 1998; Lee, 2005; Choi and Lin, 2009; Kim and Niederdeppe, 2013), but also with perceived preventive efficacy toward the disease. This result echoes earlier works where perceived preventive efficacy to the disease may result in the respondents having more positive emotions (Kim and Niederdeppe, 2013). Conversely, moderate to high levels of responsibility were attributed across all stakeholders by the *less-concerned* and the *risk-majority* even when they less frequently felt positive and negative emotions during the pandemic. This may reflect these segments’ trust in the involved stakeholders and perceived importance of shared responsibility in reducing transmission, acknowledging that every entity plays their part in managing the crisis (Macnamara, 2021).

## 6. Study implications

### 6.1. Theoretical implications

This study extends research on audience segmentation of pandemic communication. Specifically, we used Slater’s (1996) recommendation of a multivariate classification which included knowledge, attitudes, and behaviors relevant to a given health domain. Existing research often relied solely on theoretical typologies (e.g., RPA framework) to perform audience segmentation during COVID-19 (e.g., Duong, 2022). In addition to risk attitudes and preventive behavior adoption, we included perceived knowledge and emotional responses felt during the pandemic as determinants of audience segmentation. The inclusion of audience profile predictors from a range of theoretically supported factors allows us to better capture differences in how various segments perceive and respond to a pandemic.

### 6.2. Practical implications

This study also has several practical implications for policymakers and practitioners of pandemic communication. Firstly, the identification of the *less-concerned* audience segment, which constitutes about one-third (32%) of the sample, may prove to be important in practice. The *less-concerned* audiences have considerably lower risk perceptions and engagement in preventive behavior, making them a primary target audience who needs an intervention. In doing so, practitioners could consider their characteristics, such as being less extroverted and less agreeable than the other audience segments, in designing tailored intervention strategies. It is also noteworthy that they have a higher tendency to seek health information from informal sources and prefer news or stories reporting conflicts which possibly indicates a higher likelihood of being more easily fatigued with constant messaging. In targeting the *less-concerned*, future strategies can use narrative messages or appeal to self-interest rather than social obligations to the community in promoting their positive attitude and behavior change (Kleitman et al., 2021).

On the other hand, the *risk-anxious* audience segment shows strong adherence to governmental recommendations due to their perceived severity of the disease. Characterized with high intensity, frequency and time spent on health information seeking, preference in mainstream media as health information sources, the *risk-anxious* also reported the highest frequency in positive and negative emotions felt during the pandemic. Future messaging targeting a *risk-anxious* segment should take a more reassuring stance to not encourage extreme behaviors such as panic buying or excessive information search and sharing.

### 6.3. Limitations and future studies

While this study provides useful insights into pandemic communication, this study is not without its limitations. First, this study focuses on Singapore, a country with high vaccination rates and compliance rates with preventive measures against COVID-19, which could be related to its unique cultural characteristics and sociopolitical environment. Thus, our findings should be cautiously interpreted and applied to different populations and pandemic contexts. Second, this study is based on a cross-sectional design that only represents one point in time in the pandemic. Due to the rapid progression of the COVID-19 pandemic, relaxation or tightening of preventive measures may change in the duration of weeks or months. Accordingly, individual perceptions and behavior may as well change with the updated measures over the course. Therefore, longitudinal studies may allow us more insights into the changes in risk perceptions and behaviors, as well as the stability of audience profiles identified over time.

Despite the reopening of economies and most international borders, the COVID-19 pandemic is still ongoing. Overexposure to messaging related to COVID-19 pandemic can lead to message fatigue. Future research could examine audience segmentation with message fatigue or examine how fatigue changes over time in audience segments. While we focused on prevention behaviors, future studies could investigate other behaviors relevant to a pandemic (e.g., panic buying and cyberchondria). Additionally, further research can investigate how various segments would perceive or act based on fake news and disinformation.

## Data availability statement

The raw data supporting the conclusions of this article will be made available by the authors, without undue reservation.

## Ethics statement

The studies involving human participants were reviewed and approved by DSO-SAF Institutional Review Board (IRB). The patients/participants provided their written informed consent to participate in this study.

## Author contributions

HK, BP, YT, and WL contributed to the conception and design of the study. YO performed the statistical analysis and wrote the first draft of the manuscript. HK and BP revised sections of the manuscript. All authors contributed to the manuscript revision, read, and approved the submitted version.

## Funding

This research was supported by the DSO National Laboratories (DSOCL21088).

## Conflict of interest

The authors declare that the research was conducted in the absence of any commercial or financial relationships that could be construed as a potential conflict of interest.

## Publisher’s note

All claims expressed in this article are solely those of the authors and do not necessarily represent those of their affiliated organizations, or those of the publisher, the editors and the reviewers. Any product that may be evaluated in this article, or claim that may be made by its manufacturer, is not guaranteed or endorsed by the publisher.

## Supplementary material

The Supplementary material for this article can be found online at: https://www.frontiersin.org/articles/10.3389/fpsyg.2023.1085208/full#supplementary-material
