# Supplementary material for: Profile identification and characterization of risk perceptions and preventive behaviors during the COVID-19 pandemic: A latent profile analysis
Source: Front Psychol. 2023 Feb 20;14:1085208. doi: 10.3389/fpsyg.2023.1085208 (PMC9986486; doi:10.3389/fpsyg.2023.1085208)
Supplement: Supplementary file 1 [file Table_1.DOCX]

Supplementary Material

# Supplementary Table

**Appendix of Measurement Items**

| Measures | Items | References |
| --- | --- | --- |
| Determinants of Audience Segmentation | | |
| Adoption of Preventive Behavior | Washed hands with soap and water | Caress et al. (2010) |
|  | Carried tissues with you |  |
|  | Avoided crowded spaces or large crowds |  |
|  | Avoided public transport at peak times |  |
|  | Used antibacterial gel |  |
|  | Worn a surgical mask |  |
|  | Avoided touching your face with your hands |  |
|  | Disinfected spaces where you live or work |  |
|  | Avoided kissing or hugging people |  |
| Perceived Knowledge | If someone asks me about COVID-19, I would have enough information to inform him or her. | Zhang et al. (2015) |
|  | I am knowledgeable about COVID-19. |  |
|  | I am well informed about the possible prevention measures regarding COVID-19. |  |
|  | I am conﬁdent about my knowledge about COVID-19. |  |
| Perceived Vulnerability | If you do not take any preventive actions against COVID-19, how likely do you think you may contract the virus within three months? | Duncan et al. (2009) |
| Perceived Severity | COVID-19 is a deadly disease. | Derived from Real & Rimal (2003). |
|  | COVID-19 has a high fatality rate. |  |
|  | COVID-19 poses a serious threat to my health. |  |
| Preventive Efficacy | I can prevent myself from contracting COVID-19. |  |
|  | Preventing COVID-19 is easy for me. |  |
| Positive Emotions | I felt hopeful, optimistic or encouraged. | Kim & Niederdeppe (2013) |
|  | I felt grateful, appreciative or thankful. |  |
|  | I felt love, closeness or trust. |  |
|  | I felt interested, alert or curious. |  |
| Sympathetic Emotions | I felt sympathy or compassion. |  |
| Negative Emotions | I felt scared, fearful or afraid. |  |
|  | I felt anxious, worried or concerned. |  |
|  | I felt sad, downhearted or unhappy. |  |
|  | I felt angry, irritated or annoyed. |  |
| Audience Segment Demographic Characteristics | | |
| Age | What year were you born? |  |
| Gender | What is your gender? |  |
| Race | What is your race? |  |
| Audience Segment Psychographic Characteristics | | |
| Big Five Personality Traits (OCEAN) | Am the life of the party. | Goldberg (1992) |
|  | Don't talk a lot. [R] |  |
|  | Feel comfortable around people. |  |
|  | Keep in the background. [R] |  |
|  | Start conversations. |  |
|  | Have little to say. [R] |  |
|  | Talk to a lot of different people at parties. |  |
|  | Don't like to draw attention to myself. [R] |  |
|  | Don't mind being the center of attention. |  |
|  | Am quiet around strangers. [R] |  |
|  | Feel little concern for others. [R] |  |
|  | Am interested in people. |  |
|  | Insult people. [R] |  |
|  | Sympathize with others' feelings. |  |
|  | Am not interested in other people's problems. [R] |  |
|  | Have a soft heart. |  |
|  | Am not really interested in others.[R] |  |
|  | Take time out for others. |  |
|  | Feel others' emotions. |  |
|  | Make people feel at ease. |  |
|  | Am always prepared. |  |
|  | Leave my belongings around. [R] |  |
|  | Pay attention to details. |  |
|  | Make a mess of things. [R] |  |
|  | Get chores done right away. |  |
|  | Often forget to put things back in their proper place. [R] |  |
|  | Like order. |  |
|  | Shirk my duties. [R] |  |
|  | Follow a schedule. |  |
|  | Am exacting in my work. |  |
|  | Get stressed out easily. |  |
|  | Am relaxed most of the time. [R] |  |
|  | Worry about things. |  |
|  | Seldom feel blue. [R] |  |
|  | Am easily disturbed. |  |
|  | Get upset easily. |  |
|  | Change my mood a lot. |  |
|  | Have frequent mood swings. |  |
|  | Get irritated easily. |  |
|  | Often feel blue. |  |
|  | Have a rich vocabulary. |  |
|  | Have difficulty understanding abstract ideas. [R] |  |
|  | Have a vivid imagination. |  |
|  | Am not interested in abstract ideas. [R] |  |
|  | Have excellent ideas. |  |
|  | Do not have a good imagination. [R] |  |
|  | Am quick to understand things. |  |
|  | Use difficult words. |  |
|  | Spend time reflecting on things. |  |
|  | Am full of ideas. |  |
| Need for Cognition | I like to solve complex problems | Cacioppo et al. (1984) |
|  | I have difficulty understanding abstract ideas. |  |
|  | I need things explained only once. |  |
|  | I can handle a lot of information. |  |
|  | I try to avoid complex people. |  |
|  | I love to think up new ways of doing things. |  |
|  | I avoid difficult reading material. |  |
|  | I am quick to understand things. |  |
|  | I love to read challenging material. |  |
|  | I avoid philosophical discussions. |  |
| Need for Affect | If I reflect on my past, I see that I tend to be afraid of feeling emotions. | Maio & Esses (2001) |
|  | I feel that I need to experience strong emotions regularly. |  |
|  | Emotions help people to get along in life. |  |
|  | I find strong emotions overwhelming and therefore try to avoid them. |  |
|  | I think that it is important to explore my feelings. |  |
|  | I would prefer not to experience either the lows or highs of emotion. |  |
|  | I do not know how to handle my emotions, so I avoid them. |  |
|  | It is important for me to be in touch with my feelings. |  |
|  | It is important for me to know how others are feeling. |  |
|  | Emotions are dangerous – they tend to get me into situations that I would rather avoid. |  |
| Factors Associated with Audience Segmentation | | |
| Platform Preference | Official government websites Official messaging applications (e.g., Whatsapp, Telegram) Official government social media | Majid & Rahmat (2013). |
|  | International health organisation websites Health-related academic databases |  |
|  | Newspapers (Online inclusive) Television Radio Healthcare pamphlets and brochures |  |
|  | Personal feed on Facebook Personal feed on Instagram Personal feed on Twitter Personal messaging applications |  |
|  | Online forums Independent Blogs (e.g., Rice Media) Others |  |
| Frequency of Search for Health Info | How often did you search for health-related information on each platform selected in B1? | Ayers & Kronenfeld (2007). |
| Platform Use Frequency | How often did you search for health-related information on each platform selected in B1? | Ellison et al.(2007). |
| Intensity of Platform Use | This platform is part of my everyday activity. |  |
|  | This platform has become part of my daily routine. |  |
|  | I feel out of touch when I haven’t used this platform for a while. |  |
|  | I feel I am part of this platform’s community. |  |
| Perceived Importance of Information Type | Prevention and control of COVID-19 COVID-19 signs and symptoms Spread of COVID-19 in Singapore Availability of treatments for COVID-19 and its side effects Government’s advice for individuals having COVID-19 like symptoms Information about proper procedure for washing hands Information about proper ways for putting on a mask COVID-19 vulnerable groups and the level of risk COVID-19 protection products and their availability at major retail outlets Updated information about COVID-19 cluster areas in Singapore Updated information about current and future pandemic plan for Singapore Procedure for seeking treatment of suspected COVID-19 patients at clinics or hospitals Updated list of COVID-19 affected countries Updated number of COVID-19 fatalities in Singapore and other countries Updated number of COVID-19 infected cases across the world Origin of COVID-19 virus Updated number of COVID-19 infected patients who have recovered in Singapore | Majid & Rahmat (2013); Wong & Sam (2010) |
|  |  |  |
|  |  |  |
|  |  |  |
|  |  |  |
|  |  |  |
|  |  |  |
|  |  |  |
|  |  |  |
| News Frame Preferences | Consequences of the disease in terms of impact to human life, impact to the society, or impact to the economy | Shih et al. (2008) |
|  | Uncertainty of the disease in terms of cause, cure, or the possible spread |  |
|  | Actions taken against the disease including preventative measures or potential solutions |  |
|  | Reassurance stories emphasising the readiness or success of authorities in combating the disease |  |
|  | Conflict stories focusing on the clash in opinions about the disease (e.g. disputes about future measures, about the appropriateness of actions taken, about the future evolution of the diseases) |  |
|  | New evidence stories that talk about recent evidence which advance understanding of the disease (e.g. discovery of new strains of disease, new methods of transmission, new vaccines developed) |  |
| News Content Preference | Statistical information about the current progress and spread of COVID-19.  (e.g., number of infected, dead, recovered, new cases) | Quand et al. (2020) |
|  | Factual recounts and reports of the historical development and  current trends of COVID-19. (e.g., first cases, timeline of events, milestones, current reports, documentaries) |  |
|  | Scientific insights and expert opinions related to COVID-19. (e.g., medical and scientific reports, clinical trials, review of existing medical trends, expert interviews and sharing, explanations) |  |
|  | International affairs and developments related to COVID-19.  (e.g., global news and COVID-19 situation; WHO advisories; global relations; bi- and multilateral conversations about COVID-19; travel policy) |  |
|  | Policy developments and social support in Singapore.  (e.g., legislation, enforcement, ministerial addresses, nation-wide exercises, national cohesion and social policy) |  |
|  | Economic effects and trends of COVID-19.  (e.g., market and job stability; employment policies; businesses, services and trade; financial trends and support) |  |
|  | Anecdotal perspectives of past and current trends of COVID-19.  (e.g., personal stories and interviews from frontline medical officers; essential workers; policymakers) |  |
|  | Social and public life consequences of COVID-19.  (e.g, social distancing rules, cancellation and adaptation of events, social support groups, culture and sports) |  |
|  | Content related to Human Interest.  (e.g., content on everyday life, entertainment and other activities or events in the new normal) |  |
| Attribution of Responsibility | Citizens | Kim & Niederdeppe (2013) |
|  | Government |  |
|  | Healthcare workers |  |
|  | Schools |  |
|  | Workplaces |  |
